# Vitamin D Status in Pediatric Oncology Patients in Lebanon: A Comparison With Non-oncology Controls in a Case-Control Study

**DOI:** 10.7759/cureus.112921

**Published:** 2026-07-18

**Authors:** Rim Al Zohbi, Nada Sbeiti

**Affiliations:** 1 Pediatrics and Neonatology, Faculty of Medicine, Lebanese University, Beirut, LBN; 2 Pediatrics, Faculty of Medicine, Lebanese University, Beirut, LBN

**Keywords:** children, lebanon, oncology, pediatric, vitamin d status

## Abstract

Background and objective

Children diagnosed with cancer are more likely to have vitamin D problems due to compromised health before diagnosis, the disease itself, and cancer treatments. Previous studies, although limited in scope, have shown an elevated prevalence of vitamin D deficiency in pediatric oncology patients. This study aimed to describe the prevalence of vitamin D insufficiency among children with cancer in Lebanon.

Design and methods

This was an unmatched case-control study comparing vitamin D levels among children living in Lebanon diagnosed with cancer (cases) with those of children without cancer (controls) in Lebanon. Serum vitamin D levels were determined for both cases and controls. Patients were classified according to the Endocrine Society recommendations: deficiency (25(OH)D ≤ 20 ng/mL), insufficiency (25(OH)D 21-29 ng/mL), and normal levels (25(OH)D ≥ 30 ng/mL). Vitamin D status was evaluated by categorizing patients into two groups: hypovitaminosis D, defined as 25(OH)D levels < 30 ng/mL, and normal levels, defined as 25(OH)D levels ≥ 30 ng/mL.

Results

This unmatched case-control study included a total of 268 patients, with 67 oncology pediatric patients (cases) and 201 participants who were non-oncology pediatric patients (controls). The prevalence of vitamin D insufficiency and deficiency among children with cancer was 11 (16.45%) and 54 (80.6%), respectively. In contrast, among non-oncology patients, 103 (51.2%) showed vitamin D insufficiency, and 57 (28.4%) had vitamin D deficiency. There was a significant association between having cancer and vitamin D hypovitaminosis. Age was significantly associated with vitamin D levels among children with cancer, with older children having lower vitamin D levels.

Conclusion

Among children diagnosed with cancer, a high prevalence of vitamin D deficiency is observed, underscoring the significance of early detection and supplementation in this specific population.

## Introduction

Vitamin D deficiency is a significant global health problem affecting individuals of all ages. Among children globally, estimates range from 50% to 94%, indicating a substantial need for vitamin D [1]. Findings from several studies underscore the increased vulnerability of children with cancer to low vitamin D levels compared to the general population [2-4]. A study conducted by Helou et al. [4] in a pediatric hematology and oncology clinic in Virginia reported a striking 72% prevalence of hypovitaminosis among 89 pediatric cancer patients. Similarly, research involving 163 children with newly diagnosed cancer in California indicated that 65% had low vitamin D levels, with 32% falling into the deficiency category (≤20 ng/mL 25(OH)D concentration) and 33% classified as having inadequate levels (21-29 ng/mL 25(OH)D concentration) [5].

Comparative studies between pediatric cancer patients and healthy children showed mixed results. Some studies found comparable vitamin D levels between the two groups [3,6], while others reported significantly lower levels in children with cancer [2,4].

Children diagnosed with cancer face an elevated risk of vitamin D deficiency, stemming from both the disease itself and the numerous side effects induced by treatments [7]. Under normal circumstances, vitamin D binding protein (DBP), synthesized in the liver, binds to vitamin D (25(OH)D and 1,25(OH)D) and facilitates its transportation to target tissues [8]. Evidence from different studies suggests a correlation between plasma concentrations of albumin and DBP. During the disease process, the concentration of plasma DBP, akin to albumin, may decrease, reducing the body's capacity to transport vitamin D to target tissues [8]. Some chemotherapy agents may lead to hepatotoxicity and nephrotoxicity, impairing the enzymatic conversion of vitamin D to its active forms (25(OH)D2 and D3) and 1,25(OH)2D [9]. Certain cytotoxic drugs and radiotherapy induce photosensitivity, prompting children undergoing these treatments to avoid sunlight [7].

In Lebanon, recent data reveal an increasing incidence of childhood cancer, with over 433 children annually diagnosed [10]. Additionally, a recent study by Saad et al. [11] investigated the variation in the prevalence of hypovitaminosis D in the Lebanese population from 2009 to 2016. The study found that 43% of children experienced hypovitaminosis D (25(OH)D levels < 20 ng/mL), with higher rates among girls (47%) than boys (38%).

Given the substantial number of children treated for cancer who now survive into adulthood [12], along with the emerging evidence emphasizing the importance of vitamin D for health, coupled with the absence of published studies on this topic in Lebanon, it becomes crucial to investigate the vitamin D status of pediatric oncology patients in the country. This study represents the first attempt to determine the prevalence of vitamin D deficiency and insufficiency among children and adolescents diagnosed with cancer in Lebanon. It also aimed to compare vitamin D status between pediatric oncology and non-oncology patients and to identify clinical and demographic factors associated with deficiency.

## Materials and methods

Study design and population

We conducted an unmatched case-control study comparing vitamin D levels among Lebanese and Syrian children diagnosed with cancer (cases) with those of children without cancer (controls) in Lebanon. Demographic and clinical data were retrospectively reviewed from the medical records of patients admitted to Al Zaharaa Hospital University Medical Center (ZHUMC), Jnah, Lebanon, during the period ranging from 2023 to 2024. The study protocol was approved by the Institutional Review Board of ZHUMC in Lebanon (4/2024).

Cases

Cases were identified from the cancer registry database of ZHUMC in Lebanon, and details about them were included if they were Lebanese or Syrian, younger than 18 years, and had previously been diagnosed with cancer according to the International Classification of Childhood Cancer, third edition (ICCC-3), at the institution over the past two years, with either a solid tumor or a hematological malignancy.

Controls

Controls were selected from children admitted to ZHUMC over the past two years. Inclusion criteria for controls comprised Lebanese or Syrian nationality, age younger than 18 years, absence of a cancer diagnosis, and availability of documented vitamin D levels in their medical records. These children were either admitted for treatment of non-cancer-related conditions or were visiting the hospital for medical care during the same period as the cases.

Patients older than 18 years of age were excluded from both cases and controls. Additional exclusion criteria included the use of medications that could influence vitamin D levels, the presence of medical conditions affecting vitamin D status, and current intake of vitamin D supplements, multivitamins containing vitamin D, or enteral nutrition enriched with vitamin D. Additionally, control participants without documented vitamin D levels in their medical records were also excluded from the study.

Procedure of data collection

The initial step in the data collection process involved a detailed review of the medical records of pediatric patients to identify eligible participants for inclusion in the study. Both the case and control groups were recruited from ZHUMC, ensuring consistency and comparability in the study population.

In some cases, the selected patients who met our eligibility criteria were contacted by phone and invited to participate in the study. Parents whose children agreed to our request were asked to provide oral informed consent, and their children were scheduled for vitamin D testing. For controls, vitamin D levels were extracted from their medical files.

A structured Case Report Form (CRF) was utilized to extract relevant data from the medical records of enrolled participants (Appendix 1). This encompassed several variables, including demographic information such as age, gender, height, and weight, alongside medical history and the presence of any comorbidities. Moreover, details concerning prior vitamin D supplementation were documented. Furthermore, the dataset included clinical data related to oncology patients, including the type of malignancy and the duration of chemotherapy.

Measurement of vitamin D level

For vitamin D level measurements, all 25(OH)D levels were determined in the same laboratory using the same assay and were reported in ng/mL. Vitamin D was assayed using an electrochemiluminescence immunoassay (ECLIA) on the Cobas Pure system (Roche Diagnostics International Ltd., Rotkreuz, Switzerland). Internal quality control materials provided by Roche and external quality control materials from Bio-Rad Laboratories, Inc., Hercules, CA, USA, were used. The Endocrine Society recommendations were used to classify vitamin D status. According to the Endocrine Society, vitamin D deficiency is defined as a 25(OH)D concentration less than or equal to 20 ng/mL, and vitamin D insufficiency is defined as a 25(OH)D concentration of 21 to 29 ng/mL [13].

Data analysis

Statistical analysis was conducted using IBM SPSS Statistics for Windows, Version 24 (Released 2016; IBM Corp., Armonk, NY, USA). Descriptive statistics were performed to characterize the demographic and clinical profiles of the study population. Participant ages were stratified into two categories: children (1-12 years) and adolescents (13-18 years). For analytical purposes, infants aged less than one year were included in the children category. Cancer types were dichotomized into solid tumors and non-solid hematological malignancies, comprising leukemia and lymphoma cases. Duration of chemotherapy was categorised as less than one year (<1 year) and one year or more (≥1 year). Vitamin D status was evaluated by categorizing patients into two groups: hypovitaminosis D, defined as 25(OH)D levels < 30 ng/mL, and normal levels, defined as 25(OH)D levels ≥ 30 ng/mL. Additionally, patients were classified according to the Endocrine Society recommendations: deficiency (25(OH)D ≤ 20 ng/mL), insufficiency (25(OH)D 21-29 ng/mL), and normal levels (25(OH)D ≥ 30 ng/mL). Chi-square tests were used to compare vitamin D status between oncology and non-oncology participants and to assess associations between vitamin D categories and demographic or clinical variables among cancer patients. Fisher's exact test was used when the expected number of frequencies was fewer than five in a cell. Multiple linear regression analysis was performed to identify predictors of vitamin D levels among children with cancer and hypovitaminosis D. All studied variables were included in the multiple linear regression, including age (in years), gender, type of cancer, and duration of chemotherapy (as a categorical variable). Statistical significance was defined as a p-value ≤ 0.05.

## Results

Sample description

This case-control study included a total of 268 patients, with 67 oncology pediatric patients (cases) and 201 participants who were non-oncology pediatric patients (controls). There was no statistically significant difference in demographic characteristics between these two groups. Table 1 displays the demographic characteristics of our study participants. The mean age of the total population was 8.99 ± 4.95 years, with the majority of participants being female (144, 53.7%) and belonging to the child age group (185, 69%) (Table 1).

**Table 1:** Demographic characteristics of the study participants. "1" Infants aged less than one year (n = 2) were included in the children's group.

Variables	Cases (n=67)	Controls (n=201)	Total population (N=268)	X^2^	p-value
Mean age in years (±standard deviation)	7.88 (±5.16)	9.36 (±4.84)	8.99 (±4.95)		
Age groups				3.078	0.079
Children (1-12 years)^1^	52 (77.6%)	133 (66.2%)	185 (69%)		
Adolescents (13-18 years)	15 (22.4%)	68 (33.8%)	83 (31%)		
Gender				2.882	0.09
Male	37 (55.2%)	87 (43.3%)	124 (46.3%)		
Female	30 (44.8%)	114 (56.7%)	144 (53.7%)		

Among the cases, the mean age was 7.88 ± 5.16 years, with a predominance of male patients (37, 55.2%) and children aged between 1 and 12 years (52, 77.6%). In contrast, the control group had a mean age of 9.36 ± 4.84 years, with a majority of female participants (114, 56.7%) and the majority falling within the child age range (133, 66.2%) (Table 1).

**Figure 1:**
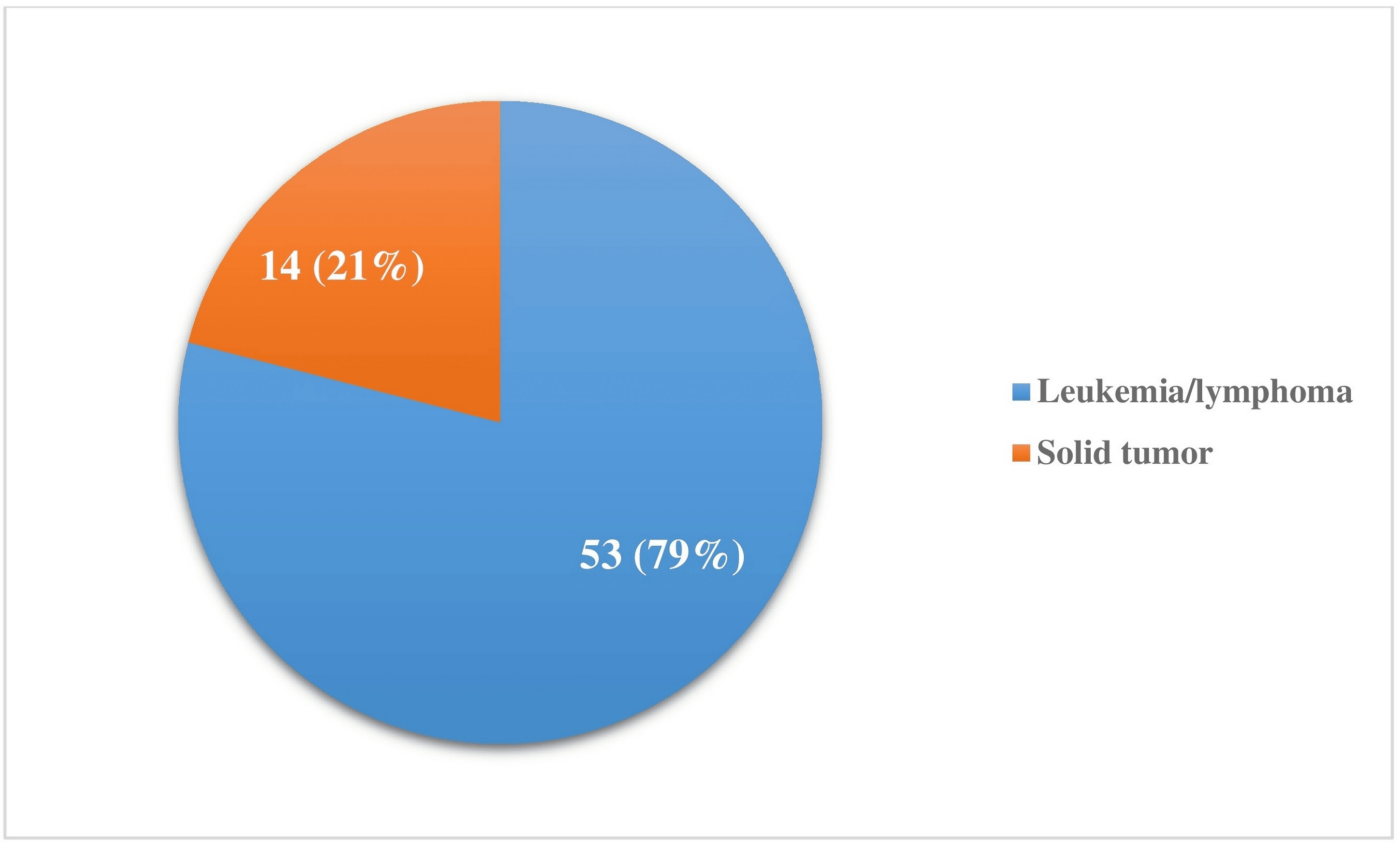
Distribution of oncology pediatric patients according to the type of cancer.

In the present study, the majority of children with cancer had leukemia or lymphoma (53, 79%), whereas only 14 (21%) had solid tumors (Figure 1).

**Figure 2:**
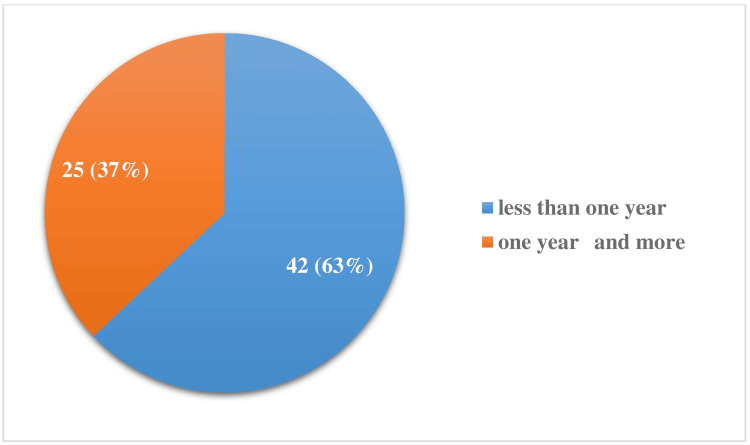
Distribution of oncology pediatric patients according to the duration of chemotherapy.

All of our cancer patients received chemotherapy, with 42 (63%) undergoing treatment for less than one year and 25 (37%) for a duration exceeding one year (Figure 2).

Status of vitamin D

In our study population, encompassing all children, vitamin D hypovitaminosis was prevalent in 84% of participants (225/268). The majority of children with cancer (65, 97%) experienced vitamin D hypovitaminosis, with only two (3%) having normal vitamin D levels. Among non-oncology children, 160 (79.6%) had vitamin D hypovitaminosis, and 41 (20.4%) had normal levels (Table 2).

**Table 2:** Prevalence of vitamin D hypovitaminosis. * Significant p-value ≤ 0.05 (Chi-square test).

	25(OH)D classification		Total	X^2^	p-value
	Vitamin D hypovitaminosis	Normal level of vitamin D			
Children with cancer	65 (97.0%)	2 (3.0%)	67	11.311	0.001*
Children without cancer	160 (79.6%)	41 (20.4%)	201		
Total	225 (84.0%)	43 (16.0%)	268		

Our study revealed a significant association between childhood cancer and the presence of vitamin D hypovitaminosis. Specifically, the proportion of vitamin D hypovitaminosis was significantly higher among children with cancer compared to those without (97% vs. 79.6%, respectively) (Table 2).

When further categorized by the level of deficiency, within the entire study population, 68 (25.4%) exhibited vitamin D insufficiency and 157 (58.6%) displayed vitamin D deficiency. Among children with cancer, 11 (16.4%) had vitamin D insufficiency and 54 (80.6%) had vitamin D deficiency. In contrast, among non-oncology patients, 57 (28.4%) showed vitamin D insufficiency and 103 (51.2%) had vitamin D deficiency (Table 3).

**Table 3:** Prevalence of vitamin D hypovitaminosis. * Significant p-value ≤ 0.05 (Chi-square test).

	25(OH)D classification			X^2^	p-value
	Vitamin D deficiency	Vitamin D insufficiency	Normal		
Children with cancer	54 (80.6%)	11 (16.4%)	2 (3.9%)	19.710	0.001*
Children without cancer	103 (51.2%)	57 (28.4%)	41 (20.4%)		
Total	157 (58.6%)	68 (25.4%)	43 (16.0%)		

Our study revealed a significant association between having cancer and the classification of vitamin D levels according to the level of deficiency. Specifically, the proportion of vitamin D deficiency was significantly higher among children with cancer compared to those without (73% vs. 44.8%, respectively) (Table 4).

**Table 4:** Distribution of vitamin D classification among children and adolescents. * Significant p-value ≤ 0.05 (Chi-square test).

		Vitamin D classification			Test	p-value
		Vitamin D deficiency	Vitamin D insufficiency	Normal		
Children	Cases	40 (76.9%)	10 (19.2%)	2 (3.8%)	17.585 (X^2^)	0.001*
	Controls	59 (44.4%)	43 (32.3%)	31 (23.3%)		
Adolescents	Cases	14 (93.3%)	1 (6.7%)	0 (0.0%)	- (Fisher-Exact)	0.105
	Controls	44 (64.7%)	14 (20.6%)	10 (14.7%)		

Comparison of vitamin D levels across same age categories

When comparing vitamin D levels among patients within the same age groups, we found a significant difference in vitamin D categories between cases and controls among children (p-value < 0.001). Vitamin D deficiency was significantly higher among cases compared to controls (40 vs. 59; 76.9% vs. 44.4%, respectively). However, among adolescents, there was no significant difference in vitamin D categories between cases and controls (p-value = 0.105).

Factors associated with vitamin D levels of children with cancer

In this study, we noticed that vitamin D deficiency was more prevalent in adolescents (14, 93.3%) compared to children (including infants) (40, 80%). However, no significant association was found between age and the severity of vitamin D deficiency among children with cancer (p-value = 0.43) (Table 4).

Although gender did not show a significant association with the severity of vitamin D hypovitaminosis (p-value = 0.54), a higher proportion of female patients with vitamin D deficiency compared to male patients (25 vs. 29; 86.2% vs. 80.6%, respectively) was observed (Table 5).

**Table 5:** Factors associated with the severity of vitamin D hypovitaminosis among children with cancer.

		25(OH)D classification		p-value
		Vitamin D deficiency	Vitamin D insufficiency	
Age groups	Children (1-12 years)	40 (80.0%)	10 (20.0%)	0.43
	Adolescents (13-18 years)	14 (93.3%)	1 (6.7%)	
Gender	Male	29 (80.6%)	7 (19.4%)	0.54
	Female	25 (86.2%)	4 (13.8%)	
Type of cancer	Leukemia/lymphoma	43 (84.3%)	8 (15.7%)	0.69
	Solid tumor	11 (78.6%)	3 (21.4%)	
Duration of chemotherapy	Less than one year	37 (90.2%)	4 (9.8%)	0.083
	More than one year	17 (70.8%)	7 (29.2%)	

While the type of cancer did not show a significant association with the severity of hypovitaminosis D (p-value = 0.69), there was a slightly higher prevalence of vitamin D deficiency among patients with leukemia/lymphoma (43, 84.3%) compared to those with solid tumors (11, 78.6%) (Table 5).

Regarding the duration of chemotherapy, no significant association was found with the severity of vitamin D hypovitaminosis (p-value = 0.08). However, children undergoing chemotherapy for less than one year showed a higher prevalence of vitamin D deficiency compared to those undergoing chemotherapy for more than one year (37 vs. 17; 90.2% vs. 70.8%, respectively) (Table 5).

Multiple linear regression

The results from the multiple linear regression analysis showed that, after adjusting for gender, type of cancer, and duration of chemotherapy, age is a significant predictor of vitamin D levels among pediatric oncology patients with hypovitaminosis D (p-value = 0.032). We found that, when age increases by one year, the vitamin D level among pediatric oncology patients with hypovitaminosis D decreases by 1.03 ng/mL on average (Table 6).

**Table 6:** Predictors of vitamin D hypovitaminosis among children with cancer.

	Beta	p-value	95% CI
Age	-0.035	0.032	-0.067 to -0.003
Gender	-0.270	0.128	-0.619 to 0.080
Duration of chemotherapy (less than 1 year: ref)	0.311	0.081	-0.039 to 0.661
Type of cancer	0.244	0.259	-0.184 to 0.661

## Discussion

Vitamin D deficiency is a global health concern associated with adverse health outcomes, including impaired bone health and immune system function [14]. Insufficient levels of vitamin D have been linked to increased risks of chronic diseases and are particularly relevant in vulnerable populations, such as pediatric patients undergoing cancer treatment [14]. To our knowledge, this study is the first study in Lebanon to assess the prevalence of vitamin D hypovitaminosis (defined as 25(OH)D levels < 30 ng/mL: vitamin D deficiency is defined as a concentration of 25(OH)D less than or equal to 20 ng/mL, and vitamin D insufficiency is defined as a 25(OH)D concentration of 21 to 29 ng/mL) among pediatric cancer patients compared to non-oncology pediatric patients in Lebanon.

In the present study, we found a high prevalence of vitamin D deficiency/insufficiency among pediatric cancer patients (65, 97%), with 11 (16.4%) exhibiting vitamin D insufficiency (25(OH)D concentration of 21 to 29 ng/mL), while 54 (80.6%) had vitamin D deficiency (≤20 ng/mL). These results were generally higher than those documented in prior studies involving pediatric oncology populations [2,15,16]. In the USA, a study [2] found in their matched case-control study of 136 children with newly diagnosed cancer and 408 children without cancer that 40% of the newly diagnosed children with cancer had vitamin D deficiency and 40% had vitamin D insufficiency according to the Endocrine Society guidelines. In Turkey, a study [15] found a prevalence of 63% of vitamin D deficiency among children with cancer (25(OH)D < 20 ng/mL). In Scotland, the prevalence of vitamin D inadequacy (defined as 25(OH)D < 50 nmol/L) was 64%, with 29% of children having vitamin D deficiency (defined as 25(OH)D <25 nmol/L). A study [16] reported a 62% prevalence of 25(OH)D <20 ng/mL in 61 pediatric cancer patients in the UK. The elevated prevalence of hypovitaminosis D observed among pediatric oncology patients in our study can be attributed to specific factors in the Lebanese context. Unlike some countries, Lebanon lacks governmental regulations mandating the fortification of milk with vitamin D. Consequently, the primary source of vitamin D for individuals in Lebanon is through cutaneous synthesis in response to sun exposure [17]. However, a significant factor influencing the increased prevalence of hypovitaminosis D in our pediatric oncology patients might be the predominant diagnosis of acute lymphoblastic leukemia (ALL) within the study patients. Numerous studies have illustrated that patients with ALL face an increased risk of developing vitamin D deficiency/insufficiency compared to children with other types of cancer [18]. Other factors include diagnostic criteria, cultural and lifestyle habits, and dietary habits [5].

Our study revealed a significant association between childhood cancer and the development of vitamin D hypovitaminosis. Specifically, the proportion of vitamin D hypovitaminosis was significantly higher among children with cancer compared to those without (65 vs. 160; 97% vs. 79.6%, respectively), indicating that children with cancer had an elevated risk of developing vitamin D hypovitaminosis. Similar results were reported by Helou et al. [4], who found that children with cancer had a significantly higher prevalence of hypovitaminosis D compared to healthy children (72% vs. 55%, respectively). Similarly, Fullmer et al. [2] discovered that children newly diagnosed with cancer had significantly lower 25(OH)D levels at diagnosis than their matched peers without cancer (22.4 ng/mL vs. 30.1 ng/mL, respectively).

As mentioned previously, several biologically plausible reasons may explain the elevated vulnerability of cancer patients to vitamin D deficiency. Sunlight, essential for vitamin D biosynthesis, is often limited for some cancer patients due to phototoxic treatments and immunosuppression. Additionally, chemotherapy-induced hepatic and renal toxicity could impede vitamin D biosynthesis. These factors may contribute to lower 25(OH)D concentrations in children with cancer undergoing treatment.

Nevertheless, it is noteworthy to highlight that several studies have documented comparable levels of vitamin D deficiency in both children with cancer and their healthy peers. In a comprehensive meta-analysis encompassing 19 studies, the prevalence of 25(OH)D deficiency in children diagnosed with hematological malignancies was observed to be similar to that in the general pediatric population, ranging from 14% to 49% [3]. Likewise, a prospective cohort study conducted by Iniesta et al. [6] compared children diagnosed and treated for cancer with healthy children. The study revealed similar plasma 25(OH)D concentrations between newly diagnosed pediatric cancer patients and their healthy counterparts, with the prevalence of vitamin D inadequacy noted at 64% among oncology patients and 63% among healthy children.

In the present study, age demonstrated a significant association with the severity of vitamin D deficiency, revealing that older children exhibited lower levels of vitamin D. Moreover, the prevalence of vitamin D deficiency was notably higher among adolescents (14, 93.3%) compared to children (including infants) (40, 80%), suggesting a correlation between older age and an elevated risk of vitamin D deficiency. These findings align with existing literature; for example, a study [2] reported a negative correlation between age and serum 25(OH)D levels, indicating a decrease in serum vitamin D levels with increasing age. According to a meta-analysis study, older age was significantly associated with an increased risk of vitamin D deficiency [3]. This trend may be attributed to shifts in dietary preferences and variations in sun exposure habits influenced by evolving daily activities and changing clothing styles as children progress in age.

With respect to gender, there was no statistically significant difference between gender and vitamin D levels, although a slightly higher proportion of female patients with vitamin D deficiency compared to male patients (25 vs. 29; 86.2% vs. 80.6%, respectively) was observed. Similar results have been reported in a study from India, where 90% of girls had vitamin D deficiency compared to 74% of boys. The increased prevalence of vitamin D deficiency among female children with cancer compared to males can be attributed to cultural practices in Lebanon, where females tend to spend more time indoors and less time outdoors. This difference in exposure to sunlight is influenced by cultural norms and societal expectations, leading to variations in outdoor activities between genders. As a result, the reduced sunlight exposure among female patients contributes to a higher incidence of vitamin D deficiency in this group compared to their male counterparts.

Similarly, the type of cancer did not have a significant association with vitamin D deficiency in our study. This result aligns with the findings of the study conducted by Genc et al. [15]. However, there is an inconsistency in the results regarding the link between various types of cancers and low 25(OH)D levels [3]. Some studies indicated that children with ALL are at a higher risk of vitamin D deficiency [18], while contrasting studies propose that children with solid tumors are more prone to having low vitamin D levels due to the intensified chemotherapy and increased use of steroids in their treatment [4,5].

Finally, it is important to note that there is a scarcity of evidence investigating potential reasons for 25(OH)D inadequacy in pediatric cancer patients. The limited number of published studies has yielded inconclusive results, as they have taken into account various variables, making it challenging to derive meaningful conclusions [3].

This study has some limitations. The cross-sectional case-control design precludes the establishment of a causal relationship between pediatric cancer and vitamin D deficiency. Therefore, the observed findings should be interpreted as associations rather than evidence of causation. Additionally, oncology patients were recruited from a single institution, which may introduce selection bias, limiting the generalizability of the findings to a more diverse population. Furthermore, the study does not extensively explore socioeconomic factors and dietary habits, which could play a role in vitamin D levels. Lack of such information may restrict the ability to fully understand the broader context of vitamin D deficiency/insufficiency among children with cancer. Finally, the limited representation of oncology patients exhibiting normal vitamin D levels, comprising only two individuals, constrains our ability to effectively compare sociodemographic characteristics between those with normal levels and those diagnosed with hypovitaminosis D. Moreover, future multicenter and prospective studies that account for seasonal variation, sunlight exposure, and nutritional intake would be valuable to further contextualize our findings and enhance their generalizability.

## Conclusions

Our study demonstrates that children with cancer are at a significantly higher risk of vitamin D inadequacy compared to healthy and non-oncology peers, with prevalence exceeding that reported in Lebanese healthy controls. Older age was associated with lower vitamin D levels, underscoring the need for age-specific considerations in clinical care.

These findings highlight the importance of routine screening and targeted vitamin D supplementation in pediatric oncology patients, ideally at admission and throughout treatment, to reduce the risk of skeletal complications and improve overall health outcomes. Larger multicenter studies are warranted to further explore contributing factors, including socioeconomic status, dietary habits, and lifestyle, to better inform preventive and therapeutic strategies. At a policy level, these findings should support the development of national screening guidelines for vitamin D deficiency in pediatric oncology patients and provide a rationale for future interventional studies evaluating the efficacy, optimal dosing, and clinical impact of vitamin D supplementation in this high-risk population.

## Appendices

Appendix 1

Children nb:

- Age: - Gender: -height:

Case Report Form

- Weight: - receiving VD supplementation: ☐ yes ☐ no - medical history/comorbidities: - presence of disease due to vitamin D deficiency: ☐ yes ☐ no - Cancer diagnosis: ☐ yes ☐ no - If yes, type of cancer: ☐ leukemia/lymphoma ☐ solid tumor

Duration of Therapy: - VD Level:
